# Developing cost-effective seismic mineral exploration methods using a landstreamer and a drophammer

**DOI:** 10.1038/s41598-017-10451-6

**Published:** 2017-09-04

**Authors:** Alireza Malehmir, Georgiana Maries, Emma Bäckström, Monika Schön, Paul Marsden

**Affiliations:** 1 Department of Earth Sciences, Uppsala University, Uppsala, Sweden; 2 Nordic Iron Ore AB, Ludvika, Sweden

## Abstract

To be fully embraced into mineral exploration, seismic data require to be acquired fast, cheaper and with minimum environmental impacts addressing also the often brown-field highly noisy environment where these surveys are employed. Since 2013 and through a number of case studies, we have been testing a newly developed for urban environment, digital-based 240 m long, seismic landstreamer for mine planning and mineral exploration purposes. Here, we present a pilot study examining the potential of the streamer for deep targeting a known, down to approximately 850 m depth, iron-oxide mineralization in the Bergslagen mineral district of central Sweden. Combined streamer (100-3C-MEMS (micro-electromechanical system), 2–4 m spacing) and 75 wireless recorders (mixed 10 Hz and MEMS, 10 m spacing) were used. A Bobcat-mounted drophammer, 500 kg, was used to generate the seismic signal. Within 4 days, approximately 3.5 km of seismic data using 2–10 m source and receiver spacing were acquired. Reflection data processing results clearly image the mineralization as a set of strong high-amplitude reflections and likely slightly extending beyond the known 850 m depth. This is encouraging and suggests such a cost-effective exploration method can be used in the area and elsewhere to delineate similar depth range iron-oxide deposits.

## Introduction

Economic metallic deposits have usually strong seismic contrast^1, 2^, product of velocity and density, with their host rocks. Therefore, at the presence of favourable geometry, size and signal-to-noise ratio, they should be detectable using seismic methods. While seismic methods have considerably better resolution and penetration depths than other geophysical methods, the high acquisition and processing cost poses a constraint in using them routinely for mineral exploration purposes. There is therefore a high demand in reducing this cost in order the method to be established in the mineral exploration sector similar to a variety of other geophysical methods. Decrease in receiver cost and higher quality sensors have allowed some contributions but on the source side this is still an issue.

**Figure 1:**
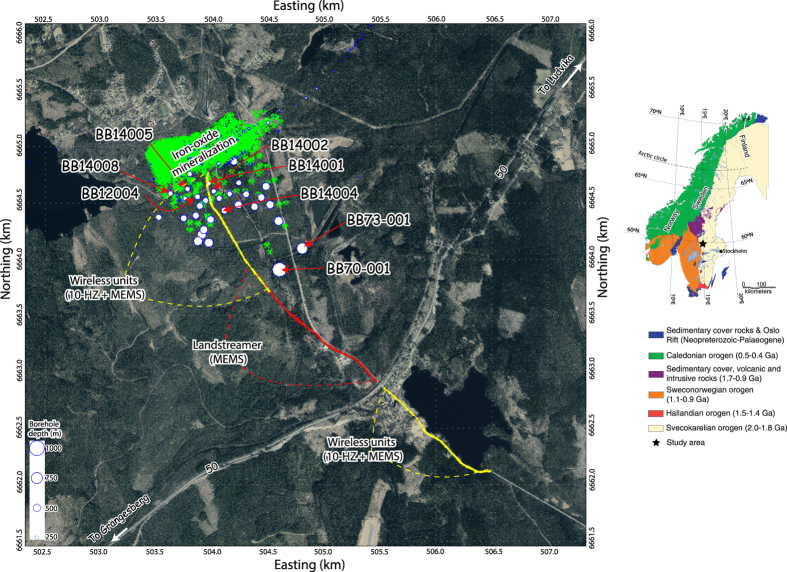
Aerial photo of the Blötberget-Ludvika mining area in central Sweden, and the location of the seismic profile (yellow and red lines). The profile was acquired using a combination of wireless recorders (yellow points) at its both ends and the landstreamer (red points) in between. Green symbols show vertical projection (to the surface) of the known mineralization, two distinct hematite and magnetite mineralized zones dipping at about 40° to the southeast and striking NE-SW. Various size circles show existing boreholes six of which (e.g., BB12004) have so far been downhole logged. The aerial photo is provided as raster image by Lantmäteriet GSD-Ortofoto 1 m (© National Land Survey, i2012/921) through Uppsala University GET (Geo Extraction Tool). The outset figure shows a simplified geology of the Scandinavia (modified from Malehmir *et al*.^19^ and produced using the Generic Mapping Tools (GMT) V4.5.0 (https://www.soest.hawaii.edu/gmt/)).

Within an on-going project involving petrophysical, geological and geophysical studies, we have examined the potential of a newly developed, for urban environment, MEMS-based seismic landstreamer^3^ for cost-effective mine planning and mineral exploration at two sites in Finland^4^ and Sweden (this study, Fig. 1), respectively. Seismic landstreamers are not new and have been used since the 80s for mainly urban or in general near-surface (>100 m) applications^5–12^. The choice of the streamer was justified in this study due to the road accessibility, and the possibility of high-voltage power cables, railroad and noise contamination if the conventional geophone-type sensors were used instead^13^. Earlier reflection seismic studies^14, 15^ targeting similar types of commodities (iron-oxide mineralization) in the region, Bergslagen mineral district of central Sweden, as the one in this study further encouraged us to take this initiative. Downhole-logging investigations including full-waveform sonic and laboratory density measurements were conducted^16^ prior to this study suggesting iron-oxide deposits (magnetite and hematite) in the study area should seismically be detectable. Therefore, with the main objectives of (1) delineating the known mineralization using the seismic landstreamer and (2) checking the penetration depth provided by a readily accessible and cheap Bobcat-mounted drophammer, we conducted a pilot seismic survey in October 2015 within 4 days and using 4 persons only. Here, we present how the data acquisition was carried out and encouraging results obtained.

## Blötberget iron-oxide deposit

The study area, Blötberget, hosting iron-oxide apatite-bearing ore bodies, is the target of a number of innovative exploration experiments currently on-going^16, 17^. We have a great level of knowledge on the mineralization and its 3D geometry, also a wealth of geophysical and downhole-logging data^16^. Important for this study are: (1) there is currently no mining operation although there are plans to reopen the mine, (2) the mineralization is dominantly magnetite but there are horizons where hematite is rich or present notably, and (3) the indicated intact ore is approximately 45 Mt (economic reserves) and is known to extend at least to 850 m depth. The mining operation stopped in year 1979 with most of the mining taking place at approximately 240 m depth at the time of closure.

The mineralization occurs within the inliers of volcano-sedimentary rocks as sheet-like bodies moderately dipping towards southwest down to 850 m depth after which it is unclear if it continues deeper or pinches out due to faulting of folding, or simply dies out. Two sets of distinct mineralization occur, on average approximately 30–50 m apart, with thicknesses varying from a few meters to sometimes over 30 m of magnetite-hematite. Sequences of granitic-pegmatitic rocks occur within the mineralization (likely concordant with the foliation).

## Downhole physical property logging

**Figure 2:**
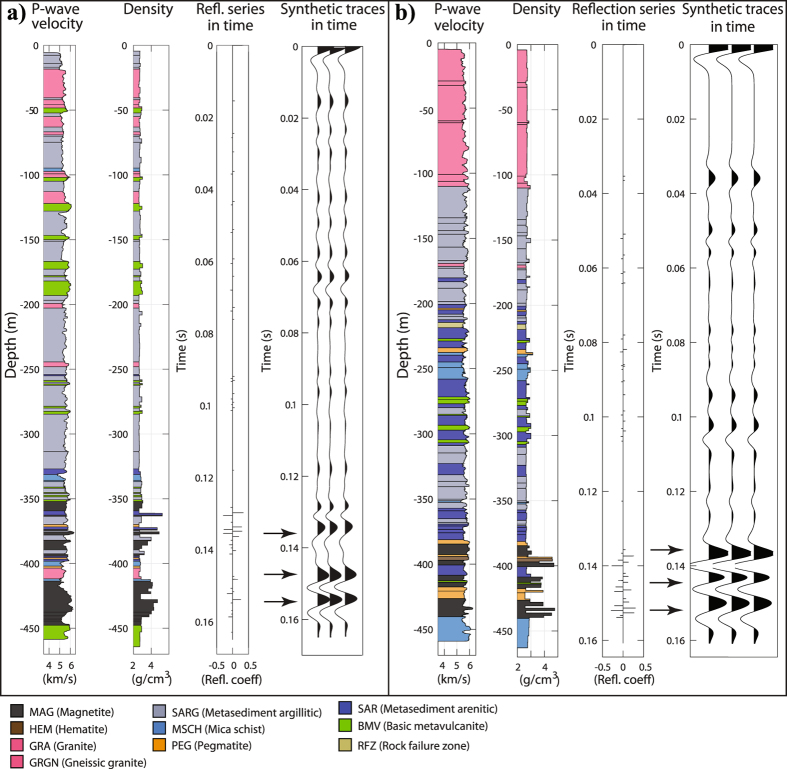
Example downhole sonic logging and laboratory density measurements from two boreholes (**a**) BB14005 and (**b**) BB14002 (for locations see Fig. 1) intersecting the mineralization at 400–450 m depth suggesting, based on 1D synthetic seismograms, a strong seismic signal can be expected from the iron-oxide deposits. Modified from Maries *et al*.^16^.

Since 2015, we have downhole logged six boreholes (Fig. 1, BB12004, BB14001, BB14002, BB14004, BB14005 and BB14008) using full-waveform triple sonic, natural gamma, magnetic susceptibility, formation resistivity, fluid conductivity and temperature. The two deepest holes, BB70-001 and BB73-001 are too slim (32 mm) to be logged. In addition to these, density measurements were conducted on core samples at every 1–3 m allowing studying seismic response of the mineralization using synthetic 1D seismograms. Figure 2 shows two examples and why we anticipate reflection seismic method to be suitable for deep targeting the iron-oxide mineralization at the site because of the high impedance contrast of the deposits with their surrounding host rock. Hematite and magnetite in fact show low resistivities and only on the order of 500–1000 ohm-m, which is not so significant to justify electric- or electromagnetic-based methods to be superior even when ignoring their limited depth resolution. Maries *et al*.^16^ detail the physical property studies and how the results can be correlated with RQD and other types of measurements.

## Seismic data acquisition and processing

**Figure 3:**
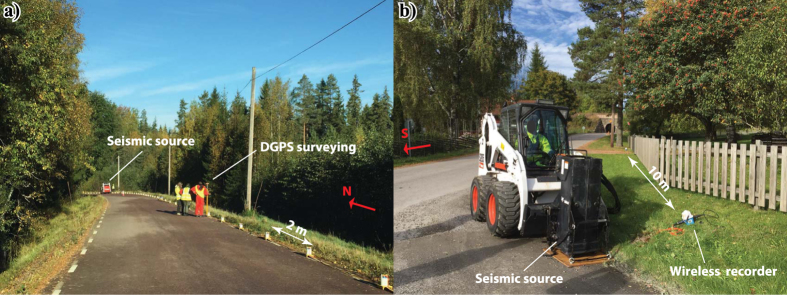
Example field photos taken during the data acquisition (October 2015) from (**a**) the seismic landstreamer, and (**b**) the Bobcat-mounted drophammer (500 kg) used in combination with 75 wireless recorders. Fixed array geometry was used while recording the data along the streamer. The streamer then moved to a new position (200 m forward) with 40 m overlap from the previous position. Main portion of the profile was on forest’s gravel roads. Photos by Alireza Malehmir.

The seismic data acquisition started after a quick site reconnaissance upon our arrival. Because of a major and high-traffic road running on the southern part of the profile (Fig. 1), it was decided to cover the southern part of the road using 51 wireless recorders connected to 10 Hz geophones and 24 recorders connected to MEMS sensors near the road (also railroad) and at where a small village is present; high electromagnetic noise was anticipated, hence justifying using the MEMS-based wirelesses. The streamer, totally 240 m long, comprising of 5 segments each containing 20 MEMS-based sensors were used right after the road on the northern side of the wireless segment (Fig. 3a). One of the streamer segments contained 20 sensors 4 m apart and the remaining four segments had 2 m sensor spacing. A Bobcat-mounted drophammer (500 kg) was used as the seismic source (Fig. 3b). The acquisition started from the southern-end of the profile with 3 shot records made at each receiver location and then progressed towards the streamer part on the northern side of the road. When shots were made at the last sensor of the streamer, the streamer was moved about 200 m towards the north to a new position while the wireless recorders south of the road kept fixed. The shooting-recording at every new streamer receiver position was then done and again the streamer was moved 200 m forward. The 4-m segment was used for the overlapping purposes and kept usually at the southern tail of the streamer. The streamer was moved seven times after which the wireless recorders placed south of the road were moved to the northern-end of the profile (Fig. 1) to cover that part. We did not think the wireless recorders would be able to record signal further than 1.5–2 km offset and wanted to make sure large offsets are also available on the northern part of the profile. Data recording then continued with the streamer twice moved more towards the north and then shots were made at the new position of the wireless recorders while the streamer then kept fixed at its last position. This way wireless recorders were employed on both southern- and northern-ends of the profile.

Wireless recorders were placed at every 10 m and partly overlapped the last position of the streamer on the northern side of the profile (Fig. 1). GPS times of the hits registered on the streamer data were used to extract the data from the wireless recorders operating in an autonomous mode during the whole survey. These data were then merged together and the three repeated shot records vertically stacked to improve the signal-to-noise ratio. To avoid a total failure, 23 explosive shots (charges ranging from 50–100 g) hand drilled into glacial tills down to 50–80 cm were also recorded in the middle of the profile. All receiver positions were surveyed using an accurate DGPS system to provide high-precision geodetic positioning. In the middle of the profile, accurate DGPS data could not be obtained because of the dense forests therefore some of the coordinates had to be interpolated thanks to the fixed and known spacing of the sensors in the streamer. For the quality control and further corrections, LiDAR elevation data publically available in Sweden were used. Overall we anticipate some inaccurate geodetic positioning on the order of 50 cm in some part of the profile. The whole data acquisition including mob/demob and geodetic surveying took 4 days. In total 533 shots and 90,587 traces (after the vertical stacking) were generated. Also in total, 1049 receiver locations/pegs (900 from the streamer, 75 wireless recorders south of the road and 74 recorders on the northern-end of the profile) were used. A nominal stacking fold of 40 was obtained when wireless data were also considered. Table 1 summarizes the main seismic data acquisition parameters.

**Table 1:** Main seismic data acquisition parameters, October 2015.

*Survey parameters*	
Acquisition type	Move along using the streamer (moved in total 9 times) and fixed wireless recorders (moved once)
Acquisition system	Sercel Lite 428 (GPS time stamping/sampling)
Number of receivers	100-3C-MEMS-based on the streamer and 52-1C-10-Hz and 24-3C-MEMS planted wireless recorders
Total number of receiver positions	1049 (900 from the streamer and 149 from wirelesses)
Number of shots	533 (after vertical stacking of repeated shot records)
Receiver interval	2-4 m (10 m for wirelesses)
Source interval	4 m
Maximum source-receiver offset	~2500 m using explosives and 1500 m using Bobcat drophammer
Source type	500-kg Bobcat-mounted drophammer and explosives for tests mainly (23 points)
Profile length	~3.5 km
Number of days	4 (including reconnaissance)
**Recording parameters**	
Record length	10 s (reduced to 1 s for processing)
Sampling rate	1 ms
Number of traces	90,587 (after vertical staking)
**Receiver and source parameters**	
Streamer length	In total 240 m, 4 segments 2 m sensor spacing and one segment 4 m sensor spacing
Number of sensors	100-3 C (MEMs), 52-1 C (10 Hz), 24-3 C (MEMS)
Source pattern	3 records per shot point
**Geodetic surveying**	
Method	DGPS (high precision on the order of a few centimeters), checked/corrected against LiDAR
Highest topography (a.s.l)	235 m (used also as an elevation datum)

**Figure 4:**
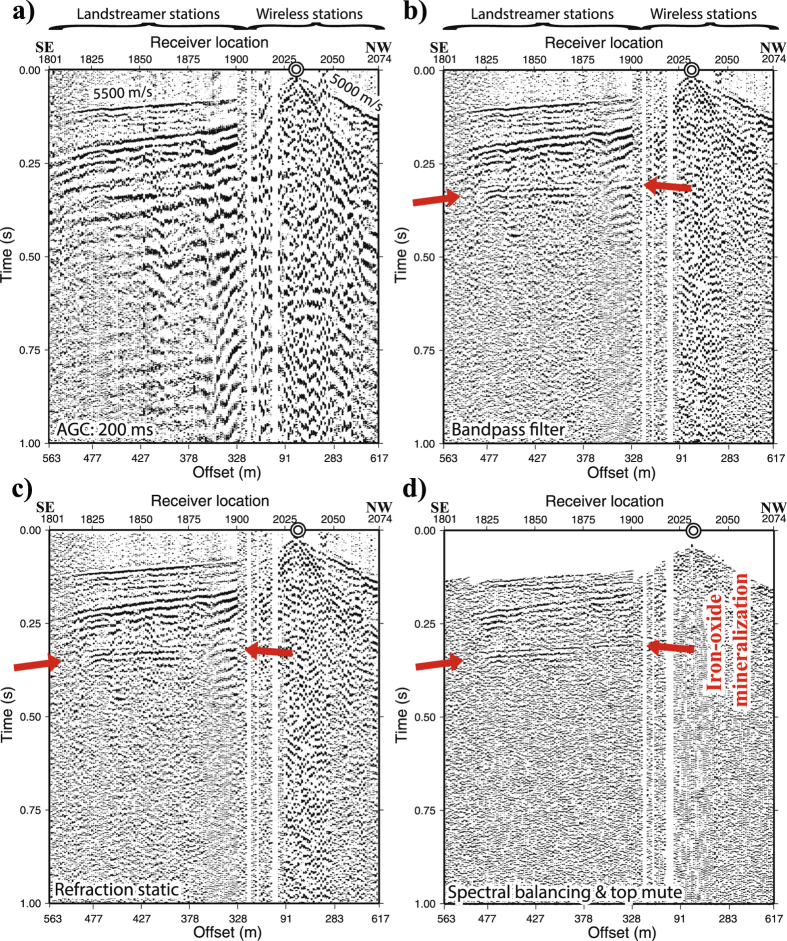
(**a**) An example of a raw shot gather from a shot fired near one of the northern wireless stations and after (**b**) bandpass filtering, (**c**) refraction static corrections, and (**d**) spectral balancing and top mute. Note the reflection marked by the red arrow and interpreted to originate from the mineralization clearly recorded in the streamer sensors. This reflection stacked constructively and contributed to the image of the mineralization.

The data processing followed a conventional poststack approach giving a focus to refraction static corrections, differentiating geophone data (to be in the acceleration domain as the MEMS data), coherent and random noise attenuation prior and after stacking, velocity analysis and NMO corrections. Figure 4 shows an example shot gather and how a shot fired near one of the wireless recorders on the northern part of the profile generated a strong reflection from the mineralization in the streamer data. The reflection is quite clear after a few processing steps.

## Results and Discussion

**Figure 5:**
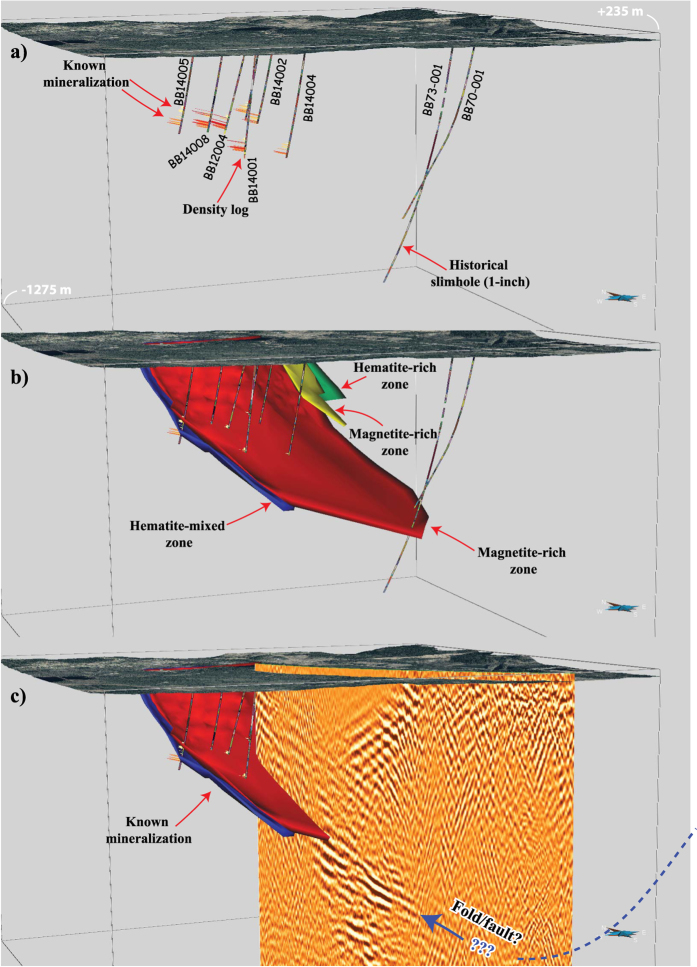
(**a**) 3D visualization of some of the boreholes downhole logged prior to the seismic survey, (**b**) with the 3D models of the ore bodies, and (**c**) unmigrated reflection seismic section of a portion of the profile with the known ore bodies. A series of strong reflections associated with the mineralization is evident down to about 1200 m depth in the unmigrated section (we estimate this to migrate to 900 m depth after migration). It is unclear why the reflections stop at this depth and if this has any exploration or geological implications.

Figure 5a shows the six deep boreholes (those labelled with the exception of BB70-001 and BB73-001) downhole logged by Maries *et al*.^16^ and visualized in 3D. The two distinct sets of mineralized zones (hematite and magnetite) are evident from the density measurements on core samples from these holes. The deepest boreholes in the area (BB70–001 and BB73-001 in Fig. 5a) intersect the mineralization at about 850 m depth as indicated by the models of the ore bodies in Fig. 5b. Figure 5c shows the reflection seismic result (unmigrated) visualized in 3D with the known mineralized bodies. The most striking feature in the section is a set of high-amplitude reflections associated with the known mineralization. It is so noticeable and bright-spot looking that it cannot be anything than from the mineralization; it is also consistent with the logging data (Fig. 2) suggesting their high acoustic impedance contrast with their host rocks. Results are encouraging and suggest even a slightly deeper continuation of the mineralization from the known depth of 850 m. Given that the section is not migrated we provide information about the actual dip and position of the reflections when migrated using a constant velocity of 6000 m/s and its apparent dip of approximately 35**°**. When migrated, the reflections would have a dip of approximately 45° and would move up approximately to 900 m depth from the 1200 m depth observed in the unmigrated section^18^. It is however unclear why the mineralization stops and whether this is a problem of depth penetration or if it reflects the actual geology; an issue remained to be further investigated. The highly swampy and wetland of Blötberget also implies attenuative near-surface conditions requiring stronger sources, an issue that require a detailed investigation and comparison with conventional plant-type receivers and explosive sources.

Nevertheless, this pilot seismic experiment and set-up appears to be sufficient for delineating such a type deposit and depth range. It is highly cost-effective (50% cheaper than convectional ones) and uses only an affordable and readily accessible seismic source; it does not require extensive reconnaissance and site planning. Only a minimum set-up was required to set up the drophammer as the seismic source and a minivan to place the acquisition system (for data quality checking). The streamer system has been used in various test sites in the Nordic countries but none of those studies, also those by others, have shown such a deep potential. This test therefore places its potential highly also for deep targeting (750–1000 m) as illustrated here. Future studies should aim at comparing the streamer data with conventional plant type sensors, checking the lateral extent of the mineralization and if the abrupt stop in the mineralization is due to geology or depth penetration problems.
